# Supplementary material for: A Pharmacokinetic and Pharmacodynamic Evaluation of the Anti-Hepatocellular Carcinoma Compound 4-N-Carbobenzoxy-gemcitabine (Cbz-dFdC)
Source: Molecules. 2020 May 8;25(9):2218. doi: 10.3390/molecules25092218 (PMC7248705; doi:10.3390/molecules25092218)

Supplementary material

Figure S1. The ^{13}C NMR of Cbz-dFdC.

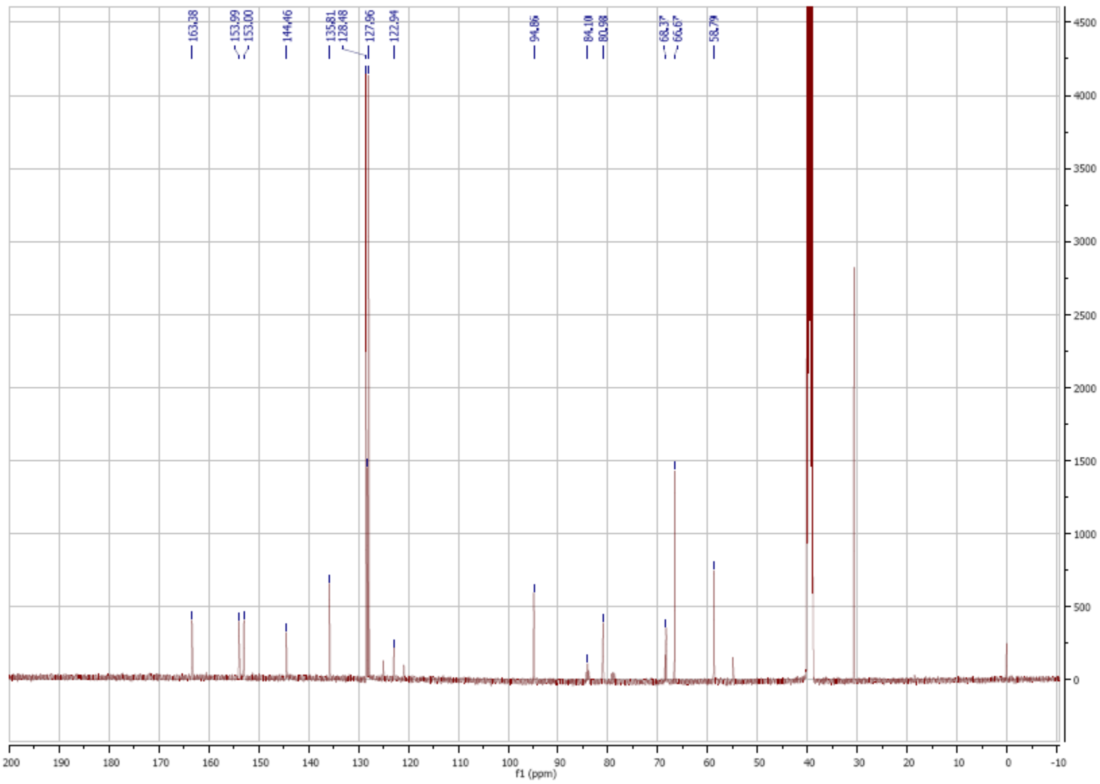

Figure S2. The ^{1}H NMR of Cbz-dFdC.

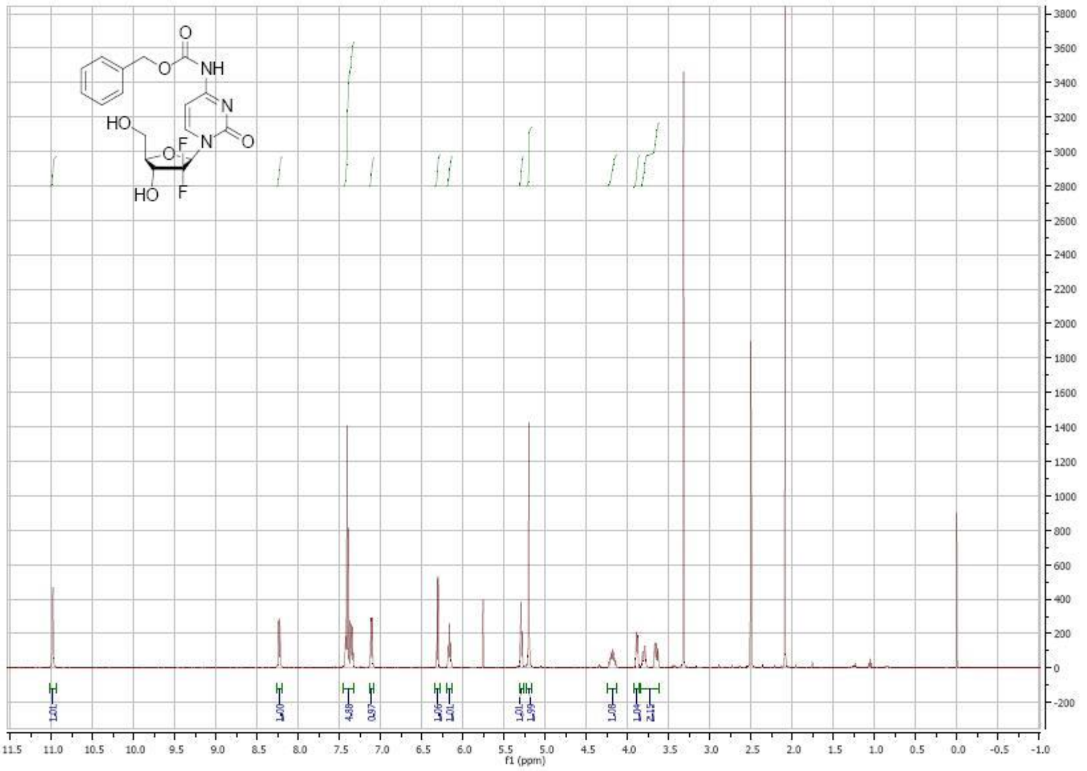

Figure S3. The Mass spectrogram of full scan of Cbz-dFdC at 200-500 m/z.

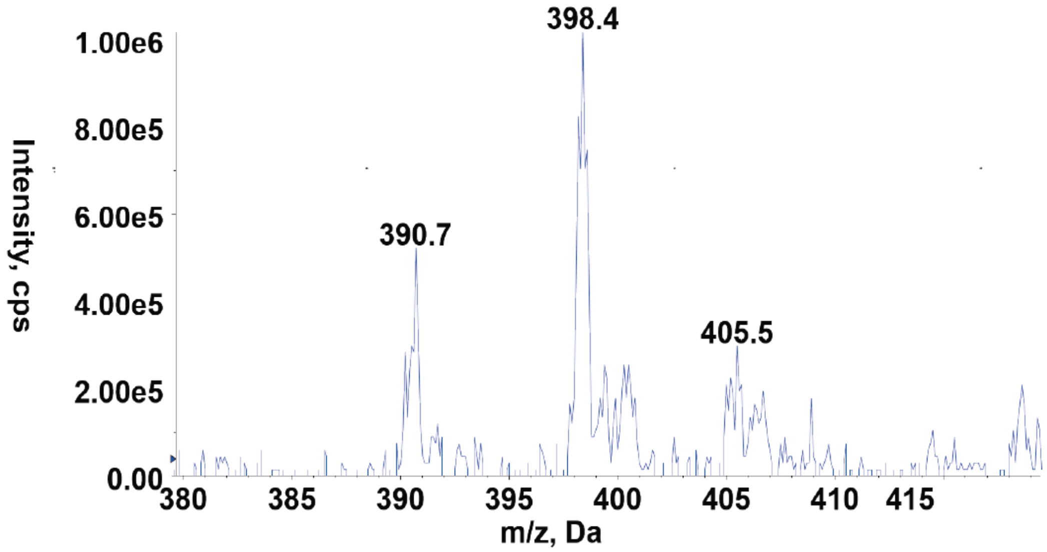

Figure S4. The Mass spectrogram of product ion of $[M+H]^+$ of the Cbz-dFdC.

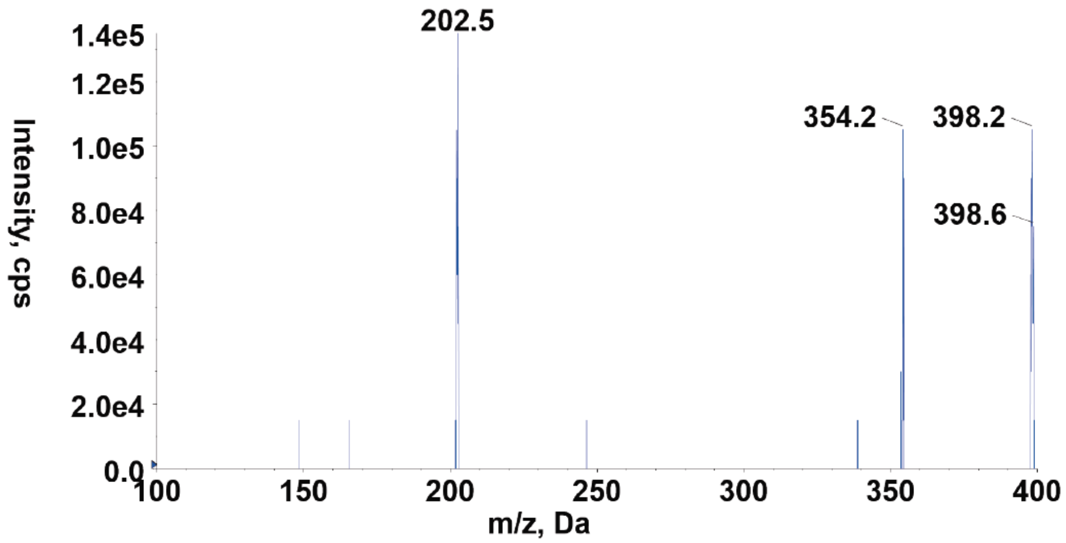

Figure S5. Chromatogram for the three substances and IS from left to right are the IS, dFdC, dFdU and the Cbz-dFdC.

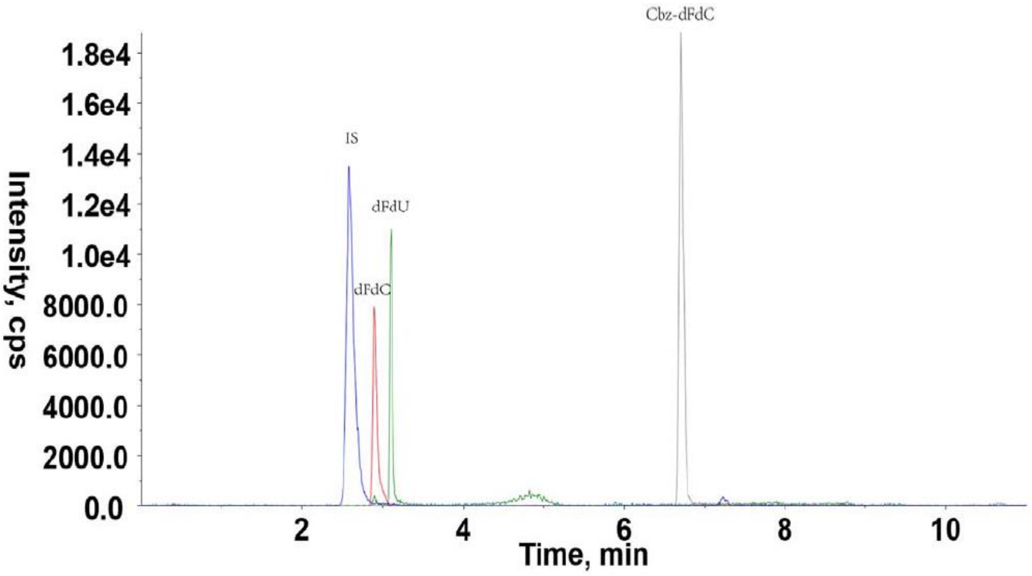

Supplement: Supplementary file 1 [file molecules-25-02218-s001.pdf]
